# Recovery of the London Hospitals

**Published:** 1924-12

**Authors:** 


### RECOVERY OF THE LONDON HOSPITALS.

### AN AGGREGATE SURPLUS.

The Hospital Economy Committee of the King's Fund has issued a report prepared from the published accounts of 116 London Hospitals showing an encouraging improvement in the finances of these institutions (Spottiswoode and Co., 2s. 6d. net). Last year there was a surplus of £227,000, that being the first time in the post-war period that the total general fund expenditure, taking the hospitals as a whole, has been met out of the revenue of the year from normal resources. It is added that, even without a legacy of £247,000, which was added to the funds, the net aggregate deficit would have been reduced from £175,000 to £20,000, a striking testimony to the progress of the hospitals towards financial stability.

### Beds and their Occupants.

Between 1921 and 1923, there was an increase in beds available of 360, but a decrease in the average occupied beds of 130, leaving a net increase in 1923 over 1913 of 1,200 available beds, and 330 average occupied beds. The disproportion between the increase in the number of available beds, and the increase in occupied beds was due to the operation of several causes, of which one of the chief was that six hospitals, aggregating 542 beds, were entirely closed to in-patients during 1923 for periods varying from three to ten months. The number of new in-patients increased between 1913 and 1921 by 19,000, while between 1921 and 1923 there was a rise of 3,600, showing a net increase in 1923 over 1913 of 22,600. The drop in the number of new out-patients in 1922, amounting to 48,000 and 73,000, as compared with 1913 and 1921 respectively, was more than recovered last year, with an increase of 85,000 over 1922, giving a net expansion of 37,000 over 1913, and 12,000 over 1921. At the same time the marked increase in out-patient attendances was maintained, last year showing an increase over 1913 of 1,452,000. The income of these 116 hospitals, apart from special distributions, increased from £1,493,000 in 1913 to £2,432,000 in 1920, and £2,595,000 in 1921. In 1922 it decreased to £2,415,000, rising again in 1923 to £2,857,000, partly in consequence of the receipt of large legacies.

### How the Difficulties were Caused.

The difficulties of the hospitals, as compared with conditions before the war, were produced by increase of expenditure and not by decrease of income, and it was satisfactory, therefore, that expenditure was considerably lower than it was three years ago. After having risen from £1,212,000 in 1913 to £2,813,000 in 1920, it fell to £2,590,000 in 1922. Last year, however, it rose by £40,000 to £2,630,000. The greater part of the fall in expenditure since 1920 has been due not to a decrease in the volume of work, but to a reduction in cost. The net aggregate deficit of the 116 hospitals amounted to £381,000, or about 14 per cent. of the expenditure for 1920 ; to £209,000, or about 7 per cent. of the expenditure for 1921 ; and to £175,000, or about 7 per cent. of the expenditure for 1922. On the other hand, last year yielded for the same hospitals a net aggregate surplus of £227,000, a figure equivalent to 9 per cent. of the expenditure. The hospitals' income from investments was £529,000, an increase of £18,000 on the previous year ; subscriptions and donations amounted to £740,000, as against £617,000 in 1922, while patients contributed £542,000 instead of £496,000.

### Quantity Statistics.

Together with this Report the Committee has issued a Memorandum on Quantity Statistics, prepared with a view to assisting and encouraging those hospitals where the importance of recording and comparing quantities consumed has not yet been fully realised. This side of hospital work, says the Introduction, is particularly important at the present time, partly because the difficulties of the hospitals are due, not to decreased income but to increase of expenditure ; partly because, at present price levels, any excessive consumption will cost far more than it did before the war ; and partly because there is reason to believe that the continued support of business men and others who have contributed to hospitals through the Combined Appeal, and in other ways, may be more readily secured if active steps are taken to develop the business side of hospital administration. "The King's Fund," it is added, "will be pleased to do what it can to assist the hospitals in any difficulties which they may experience in putting into operation the methods discussed in the memorandum, or in adapting them to the circumstances of particular institutions ; and any inquiries or suggestions will receive the most careful and sympathetic consideration of the Hospital Economy Committee."

---

### JUNIOR RED CROSS DENTAL WORK.

A modern movement, which promises to become a world-wide and permanent force, is that of the Junior Red Cross, which promotes the peace-time programme of the Red Cross among children. One of the greatest virtues of this movement is its elasticity—its capacity to adjust itself to the needs of different localities and races. The children of the Philippine Islands have specialised in the welfare of their teeth, and an amusing account is given of their achievements in *The World's Health.* According to this journal, which is published in Paris by the League of Red Cross Societies, the time may soon come when it will be considered an even greater crime to have neglected teeth than to present a dirty face to the world. In pursuit of this objective, the children of the Philippine Islands have made themselves financially responsible for a scheme as sound as it is comprehensive. Every centavo of the members' subscriptions is spent on dental work, salaries for dentists, the maintenance of dental clinics, the purchase of dental material, etc. By the end of May, 1924, there were as many as 65 dental clinics in operation, financed by the 700,141 children enrolled in the Junior Red Cross.

